# Targeting TAMs & CAFs in melanoma: New approaches to tumor microenvironment therapy

**DOI:** 10.32604/or.2025.064677

**Published:** 2025-08-28

**Authors:** Yuriy Mayasin, Maria Osinnikova, Daria Osadchaya, Victoria Dmitrienko, Anna Gorodilova, Chulpan Kharisova, Kristina Kitaeva, Ivan Filin, Valeria Solovyeva, Albert Rizvanov

**Affiliations:** 1 Institute of Fundamental Medicine and Biology, Kazan Federal University, Kazan, 420008, Russia; 2 Division of Medical and Biological Sciences, Tatarstan Academy of Sciences, Kazan, 420111, Russia

**Keywords:** Melanoma, Tumor microenvironment, Cancer-associated fibroblast (CAF), Tumor-associated macrophage (TAM), Fibroblast activation protein alpha (FAPα), Colony-stimulating factor 1 receptor (CSF1R), Clinical trials

## Abstract

Melanoma is a malignant neoplasm with a high propensity to metastasize, arising from melanocytes and contributing significantly to global morbidity and mortality. Despite the demonstrated efficacy of many immunotherapy approaches, these methods rely on direct destruction of tumor cells with minimal impact on the aggregate of nearby non-tumor cells, the extracellular matrix, and blood vessels that form the tumor microenvironment (TME). The TME is known to be heterogeneous and dynamic, exerting both antitumor and pro-tumor effects depending on the specific features and stage of carcinogenesis. TME has been shown in several studies to promote malignancy, angiogenesis, and metastasis in tumors in general and melanoma in particular. Consequently, a significant number of studies in the field of melanoma therapy have been redirected to investigate the effects of individual TME constituents, their prognostic significance for patients, and the potential of therapeutic intervention to improve overall patient survival. This review highlights novel therapeutic approaches targeting two key resident cell types in the melanoma microenvironment: tumor-associated macrophages (TAMs) and cancer-associated fibroblasts (CAFs). The review discusses their role in disease progression and summarizes the results of preclinical and clinical trials of targeted therapies against these cell types in the melanoma TME.

## Introduction

Melanoma remains a significant global health concern, with incidence and prevalence steadily increasing [1,2]. Projections indicate that the number of new cases could reach 325,000 in 2020, while the death toll is estimated to be 57,000 [3]. By 2040, melanoma incidence and mortality rates are expected to increase by 50% and 68%, respectively. Consequently, there is an urgent need for new therapeutic approaches. Over the past decade, extensive research has advanced our understanding of tumor biology, with increasing focus on the tumor microenvironment [4].

The majority of solid tumors, including melanoma, have been observed to reorganize the adjacent tissue, forming atypical tissue architecture and filling the stroma with non-resident stromal and immune cells associated with the tumor [5,6]. Melanoma cells modify the gene expression patterns of surrounding cells through soluble factors, paracrine cross-talk [7,8], extracellular vesicles and exosome [9,10], extracellular microRNA [11,12], and cell-cell contacts [13,14], extracellular matrix (ECM) restructuring [15,16], metabolites [17], and epigenetic landscape modifications [18] promoting tumor survival, immune evasion, and metastasis [19–22]. Melanoma cells produce numerous neoantigens and are highly immunogenic. As a result, various immune cells infiltrate the tumor stroma, forming the tumor immune microenvironment, with tumor-infiltrating lymphocytes (TILs) playing a key role in the antitumor response [23,24]. Due to the functional heterogeneity present within immune cell populations, the role of these cells in the tumor microenvironment (TME) remains ambiguous. However, they have the capacity to stimulate antitumor action. Among the most well-defined “anti-tumor” cell types are N1-neutrophils [25], M1-macrophages [26], activated mature dendritic cells (DC) [27,28], and natural killer cells (NK) [29]. However, some immune cells can exert suppressive effects in the TME. For example, these properties have been shown for myeloid-derived suppressor cells (MDSCs) [30], tumor-associated neutrophils (TANs) [31], suppressive phenotypes of DCs [32], for regulatory T- and B-lymphocytes (Tregs and Bregs) [33,34], and tumor-associated macrophages (TAMs). In addition to immune cells, non-immune cells, such as cancer-associated fibroblasts (CAFs) [35], adipocytes [36], keratinocytes [37], endothelial cells and their precursors [38], can also exert a special pro-tumor effect in melanoma models.

TME is a complex system in which various factors influence the progression of cancer. A key component of the TME is the altered ECM, which provides structural integrity and regulates the spatial organization of normal tissue [39]. In tumors, the ECM undergoes significant modifications, including collagen fiber rearrangement. These modifications alter matrix rigidity, limit nutrient availability, and contribute to acidosis and hypoxia [40,41]. In a previous study, the targeting of the ECM as a potential target for melanoma therapy in clinical practice was detailed [42].

Most of these components help maintain balance in the antitumor immunity cycle. However, the presence of a macroscopic tumor may suggest that the immune system is unable to effectively suppress tumor growth [43,44]. In such cases, melanoma therapy may focus on restoring balance to the antitumor immune response. This can be achieved by targeting the suppression of pro-tumor TME components or by enhancing the effector functions of specific cell populations within the tumor microenvironment [45,46].

This review aims to systematically analyze novel therapeutic approaches that specifically target two crucial cellular components of the melanoma tumor microenvironment: tumor-associated macrophages and cancer-associated fibroblasts. The primary hypothesis guiding this review is that precise modulation or depletion of TAMs and CAFs could significantly alter the tumor-promoting functions of the melanoma microenvironment, thereby inhibiting tumor growth and metastasis. By critically examining recent preclinical studies and clinical trials, the review seeks to identify promising molecular targets and evaluate their potential to enhance current melanoma therapies and patient outcomes.

## Melanoma Therapy Targeting TME and Clinical Trials

As previously mentioned, the combined interactions of the cellular and matrix microenvironment of a solid tumor ensure tumor persistence and progression, and in the particular case of melanoma. Consequently, significant cell populations within the TME, pivotal to oncogenesis and the sustenance of tumor growth, emerge as viable targets for therapeutic intervention across diverse cancer types [47]. The close and partly synergistic relationship between CAF and TAM populations in TME is well described and important for the progression of solid tumors [48]. The ratio of these cell populations within the TME is contingent upon the specific tumor type and stage. However, these two types of non-malignant cells frequently constitute a quantitatively predominant component of the stromal element in numerous types of solid tumors [49]. They are regarded as pivotal regulators of the TME [50]. CAFs secrete a pool of various cytokines and chemokines that recruit monocytes from the bloodstream into the TME, with subsequent differentiation into TAMs [51]. Additionally, their collaborative function is highlighted by their common involvement in ECM restructuring and neoangiogenesis [52]. Consequently, there is a compelling rationale for a concerted effort to collect and analyze approaches to melanoma targeted therapy that aim into CAFs and TAMs.

### Cancer-associated fibroblasts

Fibroblasts, cells of mesenchymal origin, play a pivotal role in the maintenance of connective tissue structure and function. These cells are responsible for synthesizing and secreting components of the ECM, including collagen, fibronectin, laminin, elastin, and glycosaminoglycans [53–55]. The synthesized proteins and other molecules provide mechanical support, contribute to tissue elasticity and strength, and regulate cellular processes such as growth, migration, and differentiation [56,57]. Additionally, fibroblasts play a crucial role in tissue repair by actively participating in wound healing and ECM remodeling processes [58,59]. Under normal conditions, they help maintain tissue homeostasis; however, in pathological conditions, such as chronic inflammation or oncogenesis, they can switch to an activated state, changing their phenotype [60,61].

CAFs represent a quantitatively significant component of the TME, influencing tumor growth, invasion, angiogenesis, and inflammation through a variety of mechanisms [62]. The precise mechanism of CAF formation in the tumor niche remains to be elucidated; however, studies have identified several potential factors that may play a pivotal role in this process. These include cytokines such as transforming growth factor-beta (TGF-β), platelet-derived growth factor (PDGF), fibroblast growth factor 2 (FGF2), interleukin (IL)-6, and chemokine (C-X-C motif) ligand 12 (CXCL12) [63–65], vesicular and exosomal transport products such as microRNAs and proteins [66–68], and metabolic changes involving alterations in the stroma, including hypoxia and acidosis [69,70]. In the context of melanoma, numerous aspects of the microenvironment’s formation remain to be elucidated. However, evidence suggests that CAFs might play a similarly pivotal role in disease progression as they do in more extensively studied tumor types, including epithelial cancer [71]. Recent studies have demonstrated that melanosomes, which are melanosome-specific lysosomal organelles responsible for storing melanin, possess the capacity to induce the reprogramming of normal fibroblasts into CAFs under *in vitro* conditions. This process is facilitated by the induction of proinflammatory cytokines and an augmentation in the content of specific microRNAs that are linked to the CAF phenotype [72,73].

It has been demonstrated that the CAF subpopulation, despite its inherent heterogeneity, exhibits a consensus set of characteristic features. These include potential pro-oncogenic activity, the capacity to secrete ECM components and soluble factors through the “wound healing” pathway, and the expression of specific proteins, with smooth muscle alpha-actin (αSMA) being particularly prevalent [74,75]. A critical challenge in the implementation of anti-CAF therapy is the identification of specific markers for this subpopulation of cells [76]. Typically, fibroblasts exhibit the expression of vimentin, fibroblast-specific protein 1 (FSP-1), and platelet-derived growth factor receptor alpha (PDGFRα). In the context of CAFs and certain other activated fibroblast states, αSMA, along with fibroblast activation protein alpha (FAPα), is also considered a key marker [77]. However, the heterogeneity of marker expression across different CAF populations, coupled with the ambiguity surrounding their functions within the stroma, can impede the efficacy of therapeutic targeting, leading to inconsistent outcomes.

FAPα is the most common target of choice in anti-CAF therapy due to its expression in the tumor microenvironment and its role in tumor development [78]. This protein is a type II integrin serine protease that is specifically expressed by activated fibroblasts, where it plays a pivotal role in wound healing as well as in promoting tumor growth, angiogenesis, invasion, metastasis, and immunosuppression [79]. In the absence of a tumor, FAPα exhibits enzymatic functions, including dipeptidyl peptidase and endopeptidase activities, contributing to glucose homeostasis, adipocyte metabolism, and potentially T-cell activation. However, its expression levels in normal cells are minimal [80]. Subsequent reports, however, have indicated the presence of FAPα expression in certain bone marrow-derived cells, complicating the targeting of anti-FAPα therapy [81,82]. Experimental evidence has demonstrated that FAPα, when its catalytic activity is compromised, can exert both anti-proliferative and pro-proliferative effects on tumor cells, independent of its catalytic function [83]. This duality is attributed to the activation of phosphatidylinositol-3-kinase/protein kinase B (PI3K/Akt) and matrix metalloproteinases (MMP)-2/-9 signaling pathways [84].

One of the key mechanisms underlying the non-enzymatic function of FAPα is the activation of the PI3K/Akt signaling pathway, which plays a crucial role in regulating cell proliferation, survival, and migration [85]. Recent studies have demonstrated that the expression of FAPα in tumor cells enhances the activation of this signaling cascade. Specifically, in non-small cell lung cancer cells, overexpression of FAPα significantly enhanced cell adhesion and migratory capacity, which was effectively attenuated by pharmacological inhibition of the PI3K pathway, as further confirmed by increased Akt phosphorylation observed in western blot analysis, indicating activation of PI3K/Akt signaling [86]. Notably, these effects were observed independently of the enzymatic activity of FAPα, as evidenced by experiments employing catalytically inactive mutants of the protein [84]. Additional evidence comes from models employing FAPα knockdown, where suppression of FAPα expression in oral squamous cell carcinoma cells resulted in increased phosphatase and tensin homolog (PTEN) activity and, consequently, reduced PI3K/Akt signaling, accompanied by impaired cell proliferation and metastatic potential [87]. Studies using breast cancer cell models have shown that both wild-type and catalytically inactive forms of FAPα can increase the expression and activity of MMP-2 and MMP-9, along with enhancing cellular invasiveness [84]. These findings suggest an indirect regulatory role of FAPα in MMP activation, potentially through the formation of membrane-associated multiprotein complexes involving integrins, urokinase-type plasminogen activator, and other partners that facilitate MMP secretion and activation [87]. In this context, FAPα acts as a scaffold or co-factor that promotes a pro-invasive proteolytic environment independently of its protease activity.

This was first evidenced by its identification in the invadopodia of melanoma LOX cells, where FAPα, initially termed “seprase”, was associated with gelatinolytic activity, underscoring its role in matrix degradation during melanoma invasion [88]. More recent studies have demonstrated that melanoma cells can “reprogram” normal dermal fibroblasts into CAFs expressing high levels of FAPα. Kewitz-Hempel et al. showed that melanoma-derived exosomes transfer specific microRNAs to fibroblasts, inducing their transdifferentiation into FAPα^+^ CAFs. These CAFs exhibit elevated expression of MMP-2 and MMP-9 and enhanced migratory activity [89].

Although the role of tumor cell–derived FAPα in melanoma is less well-characterized, it is well established that FAP^+^ stromal fibroblasts contribute to a tumor-promoting microenvironment by secreting pro-inflammatory and pro-invasive factors such as IL-6, IL-8, MMP-2, and MMP-9, which support melanoma progression, invasion, and therapy resistance [90]. Collectively, these findings underscore the central role of FAPα as a non-enzymatic modulator of the tumor microenvironment in melanoma, facilitating both invasive and angiogenic processes through its signaling functions.

Despite substantial evidence supporting the effective suppression of FAPα-mediated signaling, it is also important to consider potential consequences: inhibition of the PI3K/Akt signaling pathway activated by FAPα can trigger compensatory activation of the RAS/RAF/MEK/ERK cascade, as demonstrated in various cancers, and thus targeting FAPα may similarly induce alternative proliferative signaling, reducing overall therapeutic efficacy [91].

FAPα-associated approaches generally focus on either inhibiting the molecule’s proinvasive activity or targeting cells that overexpress it [78,92]. Consequently, the present analysis of CAF-targeting emphasizes FAPα and its significant therapeutic potential. Notably, comprehensive reviews on other approaches for CAF depletion in the TME have also been published recently [76,93].

**Figure 1:**
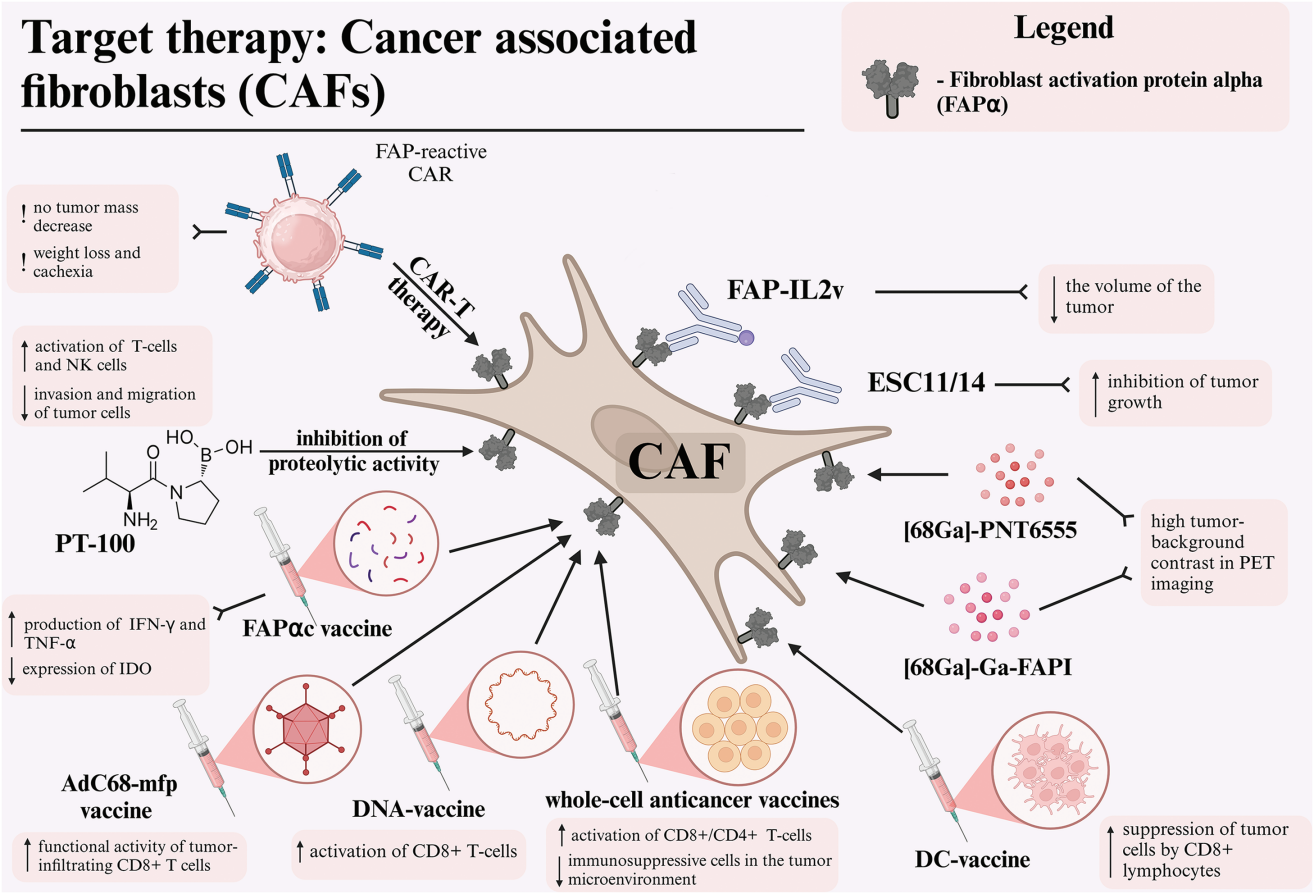
Different therapeutic approaches for targeting cancer-associated fibroblasts via fibroblast activation protein alpha (FAPα) in melanoma. Abbreviations: CAF—cancer-associated fibroblast, FAPα—fibroblast activation protein alpha, DC—dendritic cell, FAPI—fibroblast activation protein inhibitor, PET—positron emission tomography, NK—natural killer cell, CAR—chimeric antigen receptor, TNF-α—tumor necrosis factor alpha, IFNγ—interferon-gamma, IDO—indoleamine 2,3-dioxygenase. Created using BioRender. Mayasin, Y. (2025) https://BioRender.com/k27y947 (accessed on 19 Febrary 2025).

In the following section, we will be conducting a review of several key approaches to targeting CAFs via FAPα. We will also be detailing published preclinical approaches as well as clinical trial results for targeted melanoma treatment (Fig. 1).

#### Low molecular weight inhibitors

One of the pharmaceutical agents used in the anti-CAF therapeutic modality is the amino-boron dipeptide talabostat, also known as PT-100. This agent functions by targeting the inhibition of dipeptidyl peptidases, which includes the FAPα enzyme [94]. Inhibition of FAPα prevents the degradation of collagen and other components of the ECM, reducing opportunities for invasion and migration of tumor cells [95]. Additionally, inhibition of FAPα promotes the activation of T-lymphocytes and NK-cells, leading to increased production of key cytokines such as IL-2 and interferon-gamma (IFNγ), which strengthen the immune response against the tumor [96].

In a C57BL/6 syngeneic mouse model with B16-F10 melanoma, FAPα and CD26 mRNAs were found to be co-expressed in the tumor, draining lymph nodes, and spleen, yet absent in B16-F10 cells cultivated in culture *in vitro*. These findings confirm that FAPα expression is induced in TME fibroblasts in response to a malignant tumor that does not itself express this protein. It was demonstrated that the administration of talabastat at a dose of 40 μg on the second day after inoculation resulted in a substantial inhibition of tumor growth, though it did not result in complete tumor rejection. However, when tumors became palpable on days 7–8 after inoculation, the administration of the drug in doses ranging from 10 to 40 μg significantly inhibited melanoma growth, a response that was not observed in the control group. Although PT-100 demonstrated substantial activity against certain tumors in the absence of adaptive immunity, it has been established that functional T-cells are still required for tumor rejection [94].

Following the analysis of these results, a clinical trial was initiated to evaluate the efficacy of talabostat in patients with stage IV melanoma who had previously received no more than one prior line of biotherapy or chemotherapy. The drug was administered orally at a dose of 300 μg twice daily for 14 days of each 3-week cycle, with the option to increase the dose to 400 μg twice daily if well tolerated. The study enrolled 42 patients with stage IV melanoma who had previously received treatment. Efficacy was assessed in 31 patients who completed at least two cycles of therapy. According to the Response Evaluation Criteria In Solid Tumors (RECIST) criteria, treatment response was documented in two of the 31 patients (6.5%), including one patient who achieved a complete response (CR) (3.2%). The median progression-free survival was 1.5 months, while the median overall survival was 7.1 months. The most common adverse events (AEs) included edema, fatigue, hypotension, and nausea [97,98].

A phase II clinical trial was conducted to investigate the effect of the combination of talabostat and cisplatin in patients with stage IV metastatic melanoma. Patients received four 3-week cycles of cisplatin (75 mg/m^2^ on day 1) and talabostat (300 μg twice daily orally from day 2 through day 15) with dose escalation to 400 μg twice daily of talabostat depending on tolerability. Patients could continue talabostat monotherapy until progressive disease (PD) or unacceptable toxicity was established. The study enrolled 74 patients with histologically or cytologically confirmed inoperable stage IV metastatic melanoma and no more than one prior chemotherapy or biotherapy for stage IV disease. However, 32 patients (43.2%) were deemed ineligible, primarily due to nausea and vomiting associated with high-dose cisplatin, which limited the evaluation of its activity in combination with chemotherapy. The objective partial response (PR) rate was observed in 5 of the 42 patients (11.9%) who were evaluable. The duration of response ranged from 4 to 10 months, and 20 additional patients (46.5%) had stable disease (SD) for four or more cycles. The estimated median progression-free survival was 2.6 months, and the estimated overall survival was 8.5 months. The most common AEs were nausea (46%), fatigue (35%), and vomiting (34%) [99,100]. In clinical trials NCT00083239 and NCT00083252, the authors investigated the effect of talabostat on the course of disease in advanced melanoma; however, the results had not been published at the time of this article.

#### Antibodies

In a preclinical study, Fischer et al. evaluated the efficacy of radioimmunotherapy using ESC11 and ESC14 antibodies targeting FAPα in melanoma tumor stroma. In immunodeficient BALB/c^nu/nu^ mouse models with xenograft melanoma tumors (SK-Mel-187 and SK-Mel-16 lines), the authors demonstrated the efficacy of radioimmunoconjugates in inhibiting tumor growth and overall survival in the experimental group of mice compared to the control group. The study revealed that both antibodies exhibited rapid internalization capabilities; however, ^177^Lu-ESC11 demonstrated significantly higher accumulation in the tumor compared to ^177^Lu-ESC14 and the control mouse antibody vF19 [101].

Simlukafusp alfa, also known as RO6874281 (FAP-IL2v), is an immunocytokine that combines an antibody targeting FAPα with a modified IL-2 (IL-2v). It is designed to target the activation of CD8^+^ T-cells and NK cells in the tumor stroma, thereby avoiding the activation of Treg cells [102]. In the context of the B16 melanoma model, utilizing syngeneic mice of the C57BL/6 line, the studies demonstrated the binding of FAP-IL2v to FAPα in the tumor stroma, leading to the activation of interleukin-2 receptor beta-gamma chain complex (IL-2Rβγ) on effector cells, resulting in a substantial reduction in tumor volume. The combination of FAP-IL2v with anti-PD-L1 antibodies and a CD40 agonist led to a significant enhancement in survival outcomes, including long-term survival. This combination therapy exhibited minimal toxicity alongside high efficacy, underscoring its potential for clinical application [103]. A phase Ib clinical trial (NCT03875079) is currently underway to evaluate the safety and therapeutic activity of simlukafusp alfa in combination with pembrolizumab in patients with advanced or metastatic stage III-IV melanoma.

#### Dendritic cell-based vaccines

Despite over two decades of research, DC-based cancer vaccines have yet to demonstrate substantial antitumor efficacy. However, novel combination approaches are currently under investigation [104]. The antitumor efficacy of the DC vaccine is believed to be impeded by the immunosuppressive tumor microenvironment, which suppresses antigen cross-presentation and the activity of antigen-specific CD8^+^ and CD4^+^ T-lymphocytes within the tumor stroma [105]. Building on this, Gottschalk et al. developed a DC vaccine targeting both melanoma cells and CAFs expressing FAPα. The vaccine was designed to enhance the maturation of DCs by incorporating a transcription-blocking short hairpin RNA (shRNA) construct that targeted ubiquitin ligase A20, a negative regulator of the nuclear factor kappa-light-chain-enhancer of activated B cells (NF-kB) pathway. The results obtained in C57BL/6J syngeneic mouse models demonstrated that the highest antitumor efficacy against melanoma B16 was observed in DCs transduced with a vector containing a shRNA site against A20, tumor-associated antigen tyrosinase related protein-2 (TRP2), and FAPα-antigen for targeting CAF. Furthermore, the antitumor efficacy of the DC-shA20-TRP2 vaccine was comparable to that of the DC-shA20-FAPα vaccine, suggesting that targeting CAFs with FAPα vaccination can elicit antitumor effects analogous to those observed with vaccines targeting malignant cells. However, the DC-shA20-FAPα-TRP2 vaccination demonstrated the most significant antitumor activity. Mechanism studies revealed that the concurrent targeting of CAFs and tumor cells led to an increased infiltration of CD8^+^ T-cells within tumors and the induction of T-cells specific to antigens that were initially absent from the vaccine [106].

In a separate study, transfection of DCs with FAPα mRNA led to the antigen-specific activation of CD8^+^ T-cells, which were targeted towards the destruction of FAP-expressing CAFs within the tumor stroma. In a B16/F10.9 lung metastases model, the vaccination against FAPα resulted in a reduction in the number of metastases and an extension of survival time in syngeneic mice of the C57BL/6 line. Furthermore, the study demonstrated that the injection of DCs that had been transduced with FAPα mRNA led to the direct inhibition of tumor growth. This effect was further enhanced when DCs were co-transfected with TRP-2 mRNA, resulting in a synergistic increase in vaccine efficacy and a reduction in tumor progression compared to monovaccination. Antigen modification through the addition of lysosome-associated membrane glycoprotein (LAMP) signaling enhanced CD4^+^ T-cell response. This optimization improved treatment outcomes by enhancing antigen presentation through the major histocompatibility complex (MHC) class II pathway [107].

In a study by Ohshio et al., the synergistic effects of tranilast, a drug that inhibits CAFs and an antitumor dendritic vaccine, were demonstrated in C57BL/6 xenograft mouse models for various tumors, including the B16F1 melanoma line. The study demonstrated that the suppression of CAF function led to a reduction in the infiltration of immunosuppressor cells within the TME and tumor-draining lymph nodes. This, in turn, resulted in an enhancement of both cellular and humoral antitumor immune responses when administered in conjunction with DC-based vaccines loaded with tumor-associated antigens. The data demonstrated that MDSC infiltration into the TME was reduced by anti-CAF therapy, presumably due to decreased levels of CXCL12 and Prostaglandin E_2_ (PGE_2_) in tumor tissue, which enable MDSC migration into the TME. The level of a TGF-β, a critical factor in CAF-mediated transformation of naive CD4^+^ T-cells into suppressive Treg cells, was also found to be reduced due to the inhibition of CAF by tranilast and the suppression of tumor cells by CD8^+^ T-lymphocytes [108].

#### Antigenic vaccines

In two consecutive studies, Chen et al. demonstrated the therapeutic effect of therapeutic vaccines based on whole tumor cells expressing FAPα. In one case, the vaccines were made from native murine FAPα, and in the other, they were made from xenogeneic human FAPα (hFAPα). The authors showed that the vaccines exert an antitumor effect, mainly due to the activation of CD8^+^ cytotoxic T-lymphocytes and partly due to CD4^+^ T helper cells. Specifically, the study revealed that the vaccination of FAPα-expressing B16F10 melanoma cells led to a significant retardation in tumor growth and an enhancement in survival rates of C57BL/6J mice in the experimental group, as compared to the control groups in both experimental studies.

In the preceding studies, the modified tumor cells expressed FAPα, promoted the infiltration of CD8^+^ T-cells into the stroma while simultaneously reducing immunosuppressive cells in the tumor microenvironment, including MDSCs, Tregs, and M2-macrophages. This led to tumor growth suppression and the enhancement of a cytotoxic immune cell response. The most pronounced effect was observed in the B16F10 melanoma model, where both primary tumor and lung metastases were significantly inhibited. Additionally, vaccination reduced the expression of FAPα and type I collagen in tumor tissue [109].

In contrast to previous studies, a 2019 publication demonstrated that the antitumor effects were not only associated with cellular immune responses but also involved humoral immune responses. Specifically, the presence of autoantibodies against FAPα in the serum of immunized mice was observed. However, it is noteworthy that the host immune system does not typically produce autoantibodies against FAPα. Therefore, it is plausible that inactivated tumor cells expressing xenogeneic hFAPα stimulated the production of antibodies that cross-react with native FAPα expressed on CAF and host melanoma cells. Consequently, this attraction of effector cells of the immune system results in the elimination of the tumor itself and CAF in the TME [110].

Another study investigates the efficacy of a combination therapy involving a vaccine based on the FAPα core catalytic domain (FAPαc), the adjuvant CpG, and curcumin. FAPαc is a peptide containing epitopes for T- and B-cell activation and is specifically expressed by fibroblasts in tumor stroma. It effectively communicates the role of curcumin in reducing the expression of indoleamine 2,3-dioxygenase (IDO) and suppressing tumor necrosis factor alpha (TNF-α)-induced epithelial-mesenchymal transition (EMT). Both IDO and EMT are important in the context of immune response and cancer progression, respectively [111]. CpG, in turn, has been demonstrated to enhance Th1-immune responses by increasing the production of anti-tumor cytokines, such as IFNγ and TNF-α [112].

*In vitro* studies demonstrated that curcumin exhibited a dose-dependent suppression of IDO expression in B16 melanoma cells, effectively preventing the process of EMT by reducing vimentin expression and restoring E-cadherin expression. The combination therapy comprising FAPαc, CpG, and curcumin (FAPαc + CpG + curcumin) significantly enhanced survival in C57BL/6 syngeneic mice with B16 melanoma, achieving a 60% survival rate. In contrast, the groups receiving single components exhibited a mere 20% survival rate. The tumor volume was significantly smaller in the vaccinated mice than in the other groups. Histological analysis confirmed the reduction of IDO and the destruction of tumor stroma. The level of CD8^+^ T-cells producing IFNγ increased 4.4-fold compared to the control group. Curcumin complemented the effect of FAP-targeting therapy by blocking IDO and EMT, thereby enhanced the antitumor activity of the vaccine [113].

Loeffler et al. developed an oral DNA vaccine targeting FAPα. The researchers demonstrated the efficacy of the vaccine in suppressing tumor growth and metastasis by activating specific CD8^+^ T-lymphocytes. Intriguingly, the vaccine exhibited synergistic effects with chemotherapeutic anticancer drugs through FAPα-mediated inhibition of CAF and subsequent reduction in the level of intratumoral collagen type I synthesis, a crucial component of the ECM. This increase in collagen type I synthesis led to a 70% increase in the uptake of chemotherapeutic drugs by the vaccine. The most significant result was a 3-fold increase in survival of vaccinated mice receiving chemotherapy, with complete tumor rejection in 50% of the animals. This promising approach may also be applicable to melanoma [114].

A recent study by Zhang et al. evaluated an AdC68-mFAP vaccine based on an adenoviral vector expressing FAP protein, targeting FAP^+^ melanoma stromal cells. The models included Tyr::CreER, Braf^CA/+^Pten^lox+/lox+^ transgenic mice, in which melanoma formation was induced, as well as C57BL/6 syngeneic mice with transplanted B16 melanoma cages expressing murine BRAFV600E. The vaccine induced FAP-specific CD8^+^ T-cells, leading to a reduction in the number of FAPα^+^ cells, immunosuppressive cells (MDSCs and Tregs), and an alteration in the tumor cytokine environment (reduction of IL-4, IL-10, TGF-β). The results demonstrated the inhibition of tumor growth, increased survival in mice, and reduced metabolic stress. The combination of AdC68-mFAP with the AdC68-gDMelapoly vaccine, which targeted melanoma antigens, further amplified these effects, as evidenced by a reduction in tumor mass, an increase in the number and functional activity of tumor-infiltrating CD8^+^ T-cells [115].

#### CAR-T

Chimeric antigen receptor (CAR) T-cell therapy has emerged as a promising approach in cancer immunotherapy, particularly for hematological malignancies [116]. While it has shown impressive results in blood cancers, leading to FDA approval of two anti-CD19 CAR T-cell products, its efficacy in solid tumors like melanoma remains challenging [117]. In a seminal study, Tran et al. reported the administration of engineered T-cells expressing FAPα-reactive CAR to C57BL/6 syngeneic mice with transplanted tumor cells, including the melanoma line B16. According to the data obtained, melanoma cells themselves did not possess FAPα expression. However, after subcutaneous implantation into the mouse body after a certain period of time, a positive reaction to FAPα in tumor stromal tissues was shown by immunohistochemistry (IHC), probably due to the attraction of FAPα-positive stromal cells. Administration of anti-FAPα CAR-T-cells to the experimental group of mice, it was demonstrated that the antitumor effect on melanoma B16 cells was minimal and did not result in a substantial decrease in tumor mass, likely due to an insufficient tumor stroma [82].

The study reported a statistically significant decrease in weight and viability in mice receiving anti-FAPα CAR-T-cells. This decrease was not observed in mice receiving non-transduced T-cells or in mice in the negative control group. The investigation further revealed that certain stem and osteogenic cell populations, including bone marrow stem cells (BMSCs) and mesenchymal stem cells (MSCs), expressed substantial levels of FAPα, thus targeting anti-FAPα CAR-T-cells. This off-target effect resulted in weight loss and cachexia in treated mice. Thus, despite previous studies supporting anti-FAPα T-cell responses, this study underscores the limitations of FAPα as a CAR-T therapy target for melanoma and other tumors [82].

A fourth-generation CAR-T therapy targeting Nectin4/FAPα in solid tumors (excluding melanoma) is currently in clinical trials (NCT03932565). This combination of targets has previously demonstrated efficacy against metastatic colorectal cancer in mice, and the trials showed no significant limitations of earlier anti-FAPα CAR-T models. These findings suggest that combining Nectin4/FAP targeting with next-generation CARs could offer a promising strategy for treating solid tumors [118].

#### Radiotherapy and tumor tracing

Boronic acid-based DOTA-FAPIs have been shown to be potent and selective inhibitors of both soluble and membrane-associated forms of FAPα. The chelates [^68^Ga]-PNT6555 and PNT6952 have demonstrated rapid tumor uptake and high tumor-background contrast in positron emission tomography (PET), making them valuable diagnostic tools. Furthermore, the chelates [^177^Lu]-PNT6555, PNT6952, and PNT6522 exhibited significant antitumor effects in FAP-positive tumor models, with [^177^Lu]-PNT6555 demonstrating the highest therapeutic efficacy, substantiating its potential as a promising candidate for radioligand therapy [119]. Consequently, the FRONTIER study (NCT05432193) investigated the use of this drug in patients with melanoma and other FAPα-positive tumors. [^68^Ga]-PNT6555 was employed for PET diagnosis, and [^177^Lu]-PNT6555 was utilized for therapy at doses of 4, 8, and 12 GBq/cycle every 6 weeks. Among the 20 patients with FAPα-positive tumors, including melanoma, 10 received at least one cycle of therapy. The drug exhibited a favorable safety profile, with no instances of serious toxicity and no grade ≥3 AEs. The mean tumor absorbed dose was 0.16 Gy/GBq, which was lower than expected, suggesting limited retention time of the drug in tumor tissue. Despite its low doses, PNT6555 demonstrates potential as a precise and safe therapy for tumors with high FAPα expression, including melanoma, though further refinement is necessary to enhance efficacy [120,121].

In a separate study, the diagnostic potential of [^68^Ga]-Ga-FAPI, a radioligand targeting FAPα, was investigated in patients with malignant melanoma. In the case of a 41-year-old patient with metastatic melanoma of the skin, PET imaging with [^68^Ga]-Ga-FAPI exhibited higher tumor-background contrast in metastatic foci, including liver and skin metastases, compared with [^18^F]-F-FDG. The ability of [^68^Ga]-Ga-FAPI to identify lesions that were inaccessible to imaging with [^18^F]-F-FDG due to activation in the tumor stroma confirms its potential as a diagnostic tool for melanoma and other tumors with high FAPα expression in the stroma, with possible therapeutic applications [122].

The potential for targeting FAPα in melanoma stroma stems from the critical role of CAFs in establishing an aggressive tumor microenvironment. Melanoma exhibits high adaptability to the immune system, and its stroma provides a protective barrier against immune surveillance. In this context, FAPα emerges as a particularly promising target due to its predominant expression in the tumor stroma as opposed to normal tissues, which minimizes the occurrence of adverse effects. Preclinical studies have demonstrated that targeting FAPα can lead to substantial reductions in tumor volume, the suppression of metastatic potential, and enhanced survival outcomes. Clinical studies have further confirmed that a combination approach, incorporating FAPα targeting and immunotherapy modalities such as checkpoint inhibitors, can optimize therapeutic efficacy. The suppression of immunosuppression within the tumor stroma has been shown to enhance the effectiveness of other therapeutic interventions, underscoring the potential of FAPα-targeted therapy as a promising avenue for enhancing outcomes in patients diagnosed with melanoma. All clinical data (Table 1) and preclinical data (Table 2) are summarized below.

**Table 1:** Clinical researches of CAF-targeted melanoma therapy via FAPα

Target	Type of drug	Additional terms	Clinical trials ID	Phase	Disease	Status/Results	Reference
FAPα	PT-100	Cisplastin	–	II	Stage IV Melanoma	**Recruiting.** Response to treatment was recorded in 2 of 31 patients (6.5%), including one with CR (3.2%). The median progression-free survival was 1.5 months and the median overall survival was 7.1 months. The most common side effects were edema, fatigue, hypotension, and nausea.	[97,98]
			–	II	Stage IV metastatic Melanoma	**Completed.** Objective PR were observed in 5 of 42 (11.9%) patients evaluable. The duration of response ranged from 4 to 10 months. An additional twenty patients (46.5%) had SD for four or more cycles. Estimated median progression-free survival was 2.6 months; estimated overall survival was 8.5 months. The most frequent AEs were nausea (46%), fatigue (35%), and vomiting (34%).	[99,100]
			NCT00083252	II	Progressive Melanoma	**Terminated, not recruiting.** Results not published.	–
		–	NCT00083239	II	Progressive Melanoma	**Terminated, not recruiting.** Results not published.	–
	RO6874281 (FAP-IL2v)	Pembrolizumab	NCT03875079	Ib	Advanced or metastatic stage III-IV Melanoma	**Active, not recruiting.** Results not published.	–
	anti-FAPα/Nectin4 CAR-T	–	NCT03932565	I	Solid Tumors (including Melanoma)	**Active, not recruiting.** Results not published.	[118]
	[Lu-177]-PNT6555	[Ga-68]-PNT6555 for PET/CT diagnostics	NCT05432193	I	Melanoma and other FAP-positive tumors	**Active, not recruiting.** The drug demonstrated a high safety profile, with no serious toxicities (DLT) and ≥3 grade adverse events. Despite low doses, PNT6555 confirms its promise as a precise and safe therapy for tumors with high FAP expression, including melanoma, requiring refinement to improve efficacy.	[120,121]

Note: Abbreviations: FAPα—fibroblast activation protein alpha, CR—complete response, PR—partial response, SD—stable disease., DLT—dose-limiting toxicity.

**Table 2:** Preclinical researches of CAF-targeted melanoma therapy via FAPα

Target	Type of drug	Additional terms	Research object	Results	Reference
FAPα	PT-100	–	B16-F10 melanoma cell line *in vitro*; syngeneic B16-F10 melanoma cell line in C57BL/6 mice *in vivo*	Didn’t affect tumor cell viability *in vitro* but significantly inhibited melanoma growth *in vivo* without achieving full tumor rejection.	[94]
	ESC11 antibody	–	Xenografted SK-Mel-187 and SK-Mel-16 melanoma cell lines in immunodeficient BALB/c nu/nu mice *in vivo*	Demonstrated strong tumor accumulation and suppressed tumor growth, leading to extended survival in mice.	[101]
	ESC14 antibody	–		Showed low tumor accumulation and reduced efficacy compared to ESC11.	[101]
	Therapeutic vaccine based on tumor cells expressing FAPα	–	Syngeneic B16F10 melanoma cell line in C57BL/6 mice *in vivo*	Promoted CD8^+^ T-cell infiltration and reduced MDSCs, Tregs, M2 macrophages, and FAPα/collagen I levels in tumors.	[109]
	Therapeutic vaccine based on tumor cells expressing xenogeneic hFAPα	–		Induced cross-reactive antibodies that targeted both tumor and stromal FAPα^+^ cells.	[110]
	Oral DNA vaccine	–	Xenografted solid tumors in female BALB/c mice *in vivo*	Suppressed tumor growth through CD8^+^ T-cell activation and reduced collagen I, enhancing chemotherapeutic drug uptake by up to 70%.	[114]
	Anti-FAPα CAR-T-cells	–	Syngeneic B16 melanoma cell line in C57BL/6 mice *in vivo*	Caused off-target toxicity by targeting FAPα^+^ stem cells, resulting in cachexia despite reduced tumor burden.	[82]
	RO6874281 (FAP-IL2v)	Anti-PD-L1 antibody and CD40 agonist	syngeneic B16-hEGFR melanoma cell line in C57BL/6 mice *in vivo*	Reduced tumor volume and, in combination with anti-PD-L1 and CD40 agonist, improved survival with minimal toxicity.	[118]
	FAPαc + CpG + Curcumin	–	B16-F10 melanoma cell line with amniotic membrane *in vitro*; syngeneic B16-F10 melanoma cell line in C57BL/6 mice *in vivo*	Inhibited EMT markers, boosted IFNγ^+^ CD8^+^ T-cells 4.4-fold, reduced tumor size, and improved survival.	[113]
	AdC68-mFAP vaccine	–	Spontaneous melanoma in Tyr::CreER, BrafCA/^+^Ptenlox^+^/lox^+^ transgenic mice *in vivo*; syngeneic B16 melanoma cell lines in C57BL/6 mice *in vivo*	Induced FAP-specific CD8^+^ T-cell responses, decreased immunosuppressive cell populations and cytokines, and enhanced survival.	[115]
TRP2 + FAPα	DC-shA20-FAPα-TRP2	–	Syngeneic B16 melanoma cell line in C57BL/6J mice *in vivo*	Increased CD8^+^ T-cell infiltration and broadened antigen-specific responses through co-targeting of CAF and tumor cells.	[106]
	FAPα mRNA transfected DCs	DC transfected with FAPα mRNA + LAMP	Syngeneic B16/F10.9 melanoma cell line in C57BL/6 mice *in vivo*	Enhanced CD4^+^ T-cell response and antigen presentation via MHC II pathway, improving vaccine efficacy.	[107]
CAF	Tranilast	–	Syngeneic B16F1 melanoma cell line in C57BL/6 mice *in vivo*	Reduced MDSC infiltration and TGF-β expression, promoting CD8^+^ T-cell-mediated tumor suppression.	[108]

Note: Abbreviations: MDSCs—myeloid-derived suppressor cells, CAF—cancer-associated fibroblast, FAPα—fibroblast activation protein alpha, CAR-T—chimeric antigen receptor T-cells, EMT—epithelial-to-mesenchymal transition, IFNγ—interferon gamma, IL—interleukin, TGF-β—transforming growth factor beta, LAMP—lysosome-associated membrane protein, MHC—major histocompatibility complex.

### Tumor associated macrophages (TAM)

TAMs are a critical component of the TME, comparable to CAFs [123,124]. Due to the plasticity of properties and phenotypes of different populations in tumors, macrophages can exhibit both pro- and anti-tumor properties [125]. TAMs have been shown to regulate various processes, including ECM reorganization [126], inhibition of immune cells [127], secretion of anti-inflammatory factors [128], and induction of cancer cell division [129]. Additionally, TAMs have been implicated in processes such as angiogenesis and metastasis [130], antibody-dependent cell killing against cancer cells [131], and activation of lymphoid cells in both innate and adaptive immune responses [132]. Historically, the monocyte/macrophage lineage—specifically the differentiation of monocytic progenitor cells—has been identified as the primary origin of TAMs, with their emergence being traced back to the bone marrow [133]. However, subsequent research revealed that tissue-resident macrophages (TRM), which differentiate during embryogenesis from embryonic progenitor cells, can also become part of TME [134].

Alternatively activated macrophages with the M2-phenotype are believed to constitute the main component of TAM and are responsible for angiogenesis, tumor growth, and secretion of anti-inflammatory factors, while canonically activated M1-macrophages are responsible for immunostimulatory and antitumor effects [135,136]. However, in the case of skin melanoma, it is M1-macrophages that are the key component of TAM [137]. This observation challenges the prevailing two-state model for M1/M2 macrophages, suggesting instead that these states may represent intermediate phases within a broader spectrum of TAM populations [138,139]. Recent advances in single-cell RNA sequencing (scRNA-seq) and spatial transcriptomics have enabled more precise identification of TAM phenotypes and the expression profiles of individual cells within the tumor microenvironment [138]. In a recent review, Ma et al. analyzed the results of several large scRNA-seq studies of the transcriptomes of individual cells in the tumor microenvironment and identified seven TAM subpopulations based on signature genes that are found in almost all cancers. Among the subpopulations most commonly found in melanoma are: tumor-infiltrating monocytes (TIM), interferon-expressing TAMs, regulatory TAMs, and proangiogenic TAMs [140].

According to several prominent reports, TAMs in solid tumors can be also classified into four subpopulations based on core gene signatures identified by scRNA-seq: two major (C1Q^+^ and SPP1^+^) and two minor (FCN1^+^ and CCL18^+^) groups [141]. SPP1^+^ TAMs have been shown to promote tumor progression by facilitating metastasis, angiogenesis, and immunosuppression [142]. This subpopulation has been observed to enhance EMT and ECM remodeling, with high glycolytic activity and interactions with tumor cells and FAP^+^ fibroblasts contributing to metastasis and therapy resistance, particularly in liver and lymph nodes [143,144]. Conversely, C1Q^+^ TAMs have been shown to suppress T-cell activation and induce Treg differentiation via PD-L1 and IL-10 [145]. The presence of C1Q^+^ TAMs has been associated with a more favorable prognosis and a positive response to immune checkpoint blockade in certain cancers, such as melanoma, particularly when SPP1 expression is low [141]. FCN1-expressing TAMs are a type of inflammatory macrophages that facilitate angiogenesis and may act as mediators in the process of macrophage polarization, which contributes to the development of other TAM subsets and tumor progression [146]. CCL18^+^ TAMs have been observed to promote tumor growth by fostering proliferation, migration, and angiogenesis [147]. Their presence is associated with poor prognosis and facilitate immune evasion by recruiting naive T-cells that differentiate into Tregs, thereby sustaining an immunosuppressive microenvironment [148].

A recent study highlighted the distinctive features of melanoma by identifying a subset of VCAM-1^+^ TAMs using scRNA-seq in a B16F10 melanoma model. These cells formed perivascular niches, exhibited a correlation with TOX-expressing exhausted CD8^+^ T-cells, and promoted resistance to anti-CTLA-4 therapy by inducing dysfunctional T-cell activation. The gene signature of these cells was linked to poor survival in ipilimumab-treated melanoma patients [149].

A more diversified system of TAM classification provides a novel perspective on the composition of the TME. However, despite the isolation of new functional groups of macrophages, a significant part of the described subpopulations shows pro-oncogenic properties [150]. As a result, high levels of macrophage infiltration in solid tumors are rather negative prognostic markers [151]. The complexity of TAM subpopulations, including their plasticity and heterogeneity, presents a significant challenge in identifying specific targets for targeting therapy. This challenge is further compounded by the heterogeneity of CAF subpopulations, adding to the overall complexity of the system.

**Figure 2:**
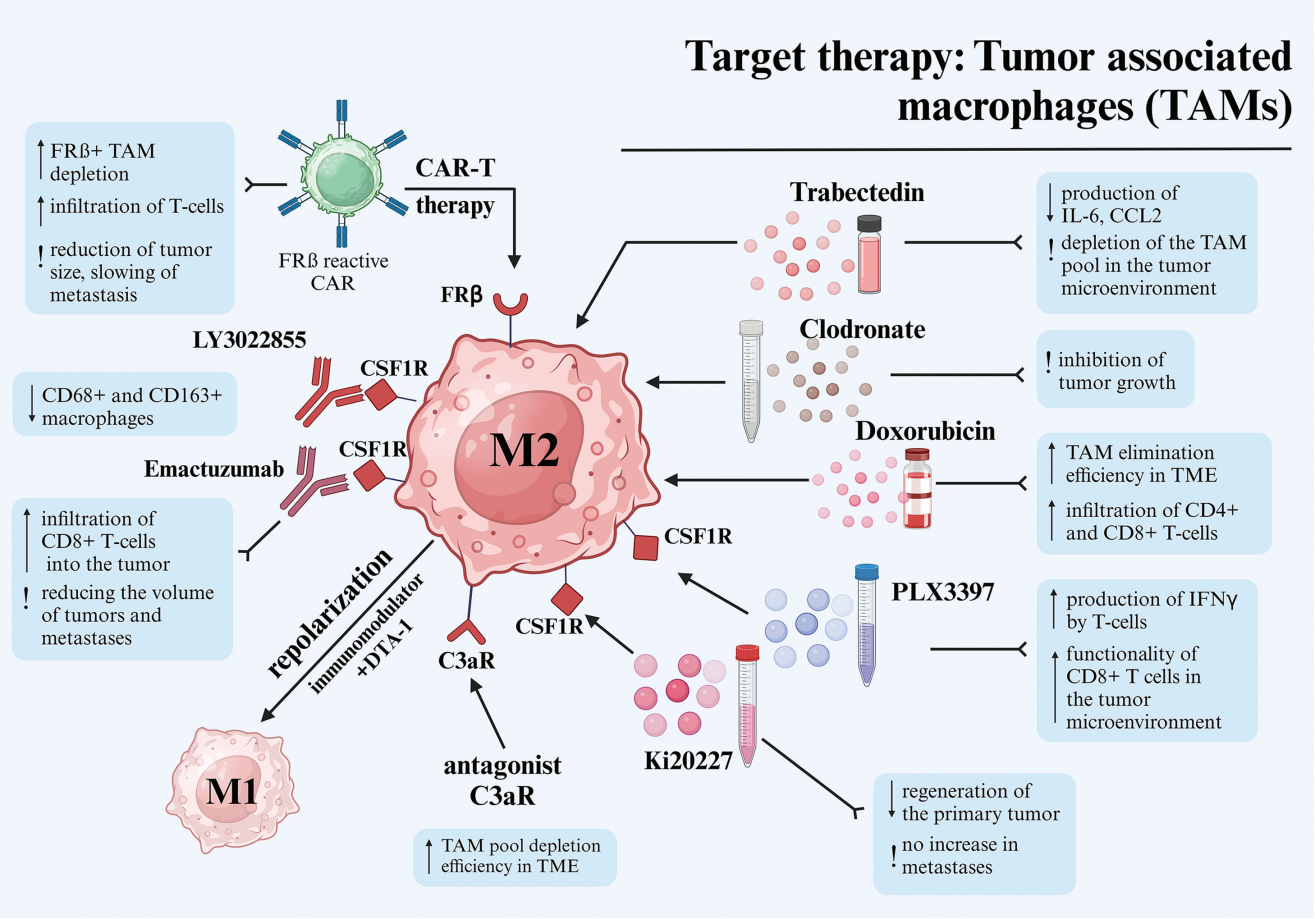
Different approaches to target therapy of tumor-associated macrophages in melanoma. Abbreviations: TAM—tumor-associated macrophages, CAR—chimeric antigen receptor, TME—tumor microenvironment, IFNγ—interferon-gamma, FRβ—folate receptor beta, C3aR—complement component 3a receptor, CSF1R—colony-stimulating factor 1 receptor, IL-6—interleukin-6, CCL2—C-C motif ligand 2. Created using BioRender. Mayasin Y (2025). https://BioRender.com/f25j752 (accessed on 19 Febrary 2025).

Below, we outline several key approaches for the targeted treatment of TAM, highlighting both published preclinical methods and the results of clinical trials of targeting melanoma (Fig. 2).

#### Low molecular weight agents

A proposed approach for targeting TAM involves depleting the pool of macrophages present within TME through exposure to various chemical agents. Trabectedin, a well-known antitumor drug derived from the shell of *Ecteinascidia turbinate*, has been shown to bind to DNA, initiating a series of processes that culminate in cell cycle disruption, altered gene transcription, and DNA repair [152]. Additionally, trabectedin inhibits the production of specific inflammatory mediators in macrophages, such as IL-6 and C-C motif ligand 2 (CCL2), which likely contributes to the depletion of the TAM pool in the tumor microenvironment [153]. The efficacy of trabectedin in combating melanoma cell lines B16-BL6 and K1735-M2 was demonstrated in mouse models C57/BL6 and C3H/HeN. This efficacy was evidenced by a substantial decrease in the density of blood vessels in the tumor stroma (for both cell lines) and a reduction in TAM accumulations (only for B16-BL6) [154].

The inhibition of CSF1R via PLX3397 inhibitor has shown a significant effect on the tumor microenvironment, as CSF1R plays a key role in the recruitment and differentiation of monocytes into TAMs that contribute to melanoma progression [155]. In one study, PLX3397 inhibitor effectively reduced TAM levels in blood and within B16F10 tumors in C57BL/6 syngeneic mice. Although PLX3397 monotherapy caused only a slight slowing of tumor growth, its combination with CD8^+^ T-cell-based immunotherapy significantly improved tumor control. This effect was accompanied by increased IFNγ production by T-cells, indicating a decrease in the immunosuppressive effect of TAM and enhanced functionality of CD8^+^ T-cells in the tumor microenvironment [156].

Erkes et al. demonstrated that in order to effectively eliminate BRAFV600E melanoma (cell lines YUMM1.7 and D4M3.A) in C57BL/6 syngeneic mouse models, it was necessary to combine the CSF1R inhibitor PLX3397 with the tumor growth inhibitor PLX51107 for subsequent TAM depletion [157]. To date, a phase I/IIa clinical trial (NCT02452424) has evaluated the efficacy of this combination therapy—CSF1R and the immunotherapeutic agent pembrolizumab—in patients with solid tumors, including melanoma. However, the study was terminated due to insufficient evidence of clinical efficacy [158]. Additionally, another phase II clinical trial assessed the efficacy and safety of the PLX3397 inhibitor in advanced acral and mucosal melanomas with receptor tyrosine kinase protein (KIT) mutations. Although the study was completed, the results remain unpublished (NCT02071940).

The therapeutic approach employed in this study involved using Ki20227, a CSF1R receptor inhibitor, to deplete macrophages post-surgical tumor resection. This strategy aimed to prevent recurrence and metastasis in a model of spontaneous metastatic uveal melanoma in RETAAD model mice. The results demonstrated that postoperative depletion of macrophages using Ki20227 led to a substantial reduction in the incidence of postoperative tumor recurrence. The study revealed a decrease in primary tumor regeneration and no increase in metastases in the Ki20227 group, resulting in a significant improvement in survival rates among the test subjects [159].

Dichloromethylene-bisphosphonate, also known as clodronate, is a hydrophilic bisphosphonate utilized for its ability to impede osteoclast activity and curtail bone resorption in cases of bone metastases. A body of research has demonstrated that clodronate can exert a direct cytotoxic effect on tumor cells by competing with ADP/ATP translocase and subsequently collapsing mitochondrial membrane potential. However, due to its hydrophilic properties, clodronate faces challenges in effectively penetrating cell membranes [160]. As a result, there is a growing interest in exploring the use of clodronate in liposomal and other modified forms to enhance its bioavailability. It has been demonstrated that liposomal forms of clodronate are efficiently absorbed by macrophages, and when administered intratumorally to TME, they induce apoptosis of TAM [161]. Treatment with clodronate-containing liposomes (clodrolip) effectively depleted TAMs in mouse models of teratocarcinoma and rhabdomyosarcoma, leading to significant suppression of tumor growth with a sharp decrease in the density of blood vessels in tumors [162].

Webster et al. applied a structural modification approach and studied bisphosphonamidate clodronate prodrug with enhanced cell penetration, with respect to direct action on melanoma with BRAFV600E mutation (cell lines SK-Mel-5 and UACC-62). The drug induced inhibition of proliferation and apoptosis of melanoma cells *in vitro*, which was confirmed by an increase in caspase-3 activity. In a SK-Mel-5 xenograft model in athymic nude mice, the administration of clodronate prodrug resulted in a significant inhibition of tumor growth in mice compared to the control group. After 19 days of treatment, the tumor volume in the experimental group was significantly reduced, while the mice’s weight remained stable, indicating the absence of significant systemic toxicity [163].

Piaggio et al. investigated a novel therapeutic strategy employing a liposomal form of clodronate (Clo-Lipo-DOTAP) for the treatment of the melanoma cell line B16/F10 in a C57BL/6JOlaHsd mouse model. This approach aimed to target the depletion of TAMs, avoid systemic toxicity, and minimize side effects on other immune system cells. The results demonstrated that Clo-Lipo-DOTAP treatment led to a substantial reduction in primary tumor volume and the number of pulmonary metastases. Immunohistochemical analysis revealed a decrease in TAMs and microvascular density within the treated tumors. Additionally, a decline in pro-angiogenic cytokines (TNF-α, IL-10, vascular endothelial growth factor (VEGF), and PDGF-bb) was observed, confirming the anti-angiogenic effect of the therapy. The reduction of TAMs and suppression of angiogenesis are hypothesized to disrupt the tumor microenvironment, thereby contributing to the inhibition of tumor growth and metastasis. However, the therapy did not affect Ki-67 expression in tumor tissues, indicating the necessity of combining Clo-Lipo-DOTAP with other treatment modalities [164].

The article by Gazzaniga et al. also investigated the effect of liposomal clodronate (Clod-Lip) on TAMs in TME using a human melanoma model of line IIB-MEL-J in xenograft athymic NIH(S)-nu/nu mice. The therapy led to a significant reduction in tumor size in mice treated with Clod-Lip compared to the control group. Subsequent analysis of the mechanism of action revealed a reduction in the infiltration of TAMs (CD11b^+^ cells), a decrease in tumor vascularization, and a decline in the expression of endothelial markers such as CD31. Additionally, the study demonstrated the effectiveness of Clod-Lip in suppressing the viability of macrophages and melanoma cells expressing monocyte chemoattractant protein-1 (MCP-1) *in vitro*. The depletion of TAMs by Clod-Lip was accompanied by a decrease in angiogenesis, as evidenced by reduced vessel density within the tumor and lower levels of proangiogenic factors, including VEGF [165].

The influence of the complement system on tumor development and the TME has been the subject of limited research. Previous studies have demonstrated the complement system’s role as a crucial component of the innate immune system, regulating inflammation and angiogenesis [166]. Contemporary research has identified the complement system as a pivotal element in cancer-related inflammation [167]. Nabizadeh et al. demonstrated that TAMs and other myeloid-derived cells can express C3aR, which is related to anaphylotoxin C3a, and can trigger a series of reactions associated with the induction of inflammation in the TME. Administration of a C3aR antagonist (SB290157) over the course of several days led to the regression of melanoma (cell lines B16-F0 and SM1WT1) in a C57BL/6J mouse model by depleting the TAM pool and promoting enhanced survival [168].

It has been demonstrated that the antitumor anthracycline antibiotic doxorubicin not only eradicates cancer cells but also exerts an influence on microenvironmental cells, such as macrophages [169]. Subsequent studies have substantiated doxorubicin’s activity against TAM [170,171]. However, achieving an effective concentration of the drug in the tumor stroma necessitates the use of targeted delivery systems, as free doxorubicin, administered intravenously, does not accumulate in the tumor, particularly in the melanoma stroma [172]. A study by Etzerodt et al. showed that lipid nanoparticles loaded with doxorubicin and conjugated with antibodies against CD163 effectively eliminated the CD163^+^ TAM population within the TME in melanoma models YUMM1.7. This efficacy was observed in both C57BL/6J syngeneic mice and in the context of spontaneous melanoma in BRAF^CA/+^, PTEN^lox4−5/lox4−5^ and Tg(Tyr-CreER^T2^) transgenic mice. Following treatment, the depleted subpopulation of suppressive CD163^+^ TAMs was replaced by an immunostimulatory subpopulation of CD11c^high^ TAMs. This resulted in enhanced infiltration of CD4^+^ and CD8^+^ T-lymphocytes into the stroma, leading to subsequent tumor regression. Following αCD163-dxr treatment, activation of effector T-cells was observed, accompanied by an increase in CD8^+^ T-cells. Thus, the selective targeting of CD163^+^ TAMs has been shown to surpass the efficacy of pan-macrophage therapies, such as CSF1R blockade, which reduces the number of antitumor T-cells, thereby limiting its effectiveness [173].

#### Monoclonal antibodies

A potential approach to targeting TAM involves the use of specific monoclonal antibodies. The primary targets of this approach are cytokine colony-stimulating factor 1 (CSF1), chemokine CCL2, and related receptors. These axes are involved in the recruitment of myelomonocytic cells to the TME and pro-tumor reprogramming. A comprehensive list of these targets can be found in a recent publication [174]. In this study, our focus will be directed towards two antibodies targeting CSF1R, which have garnered significant attention in clinical practice and have yielded notable results in published studies.

LY3022855 is a human monoclonal antibody that targets CSF1R, inhibiting its interaction with its ligands, CSF-1 and IL-34. This results in a decrease in the TAM population. A phase I/II clinical trial (NCT03101254) investigated the efficacy of LY3022855 in treating advanced melanoma with BRAF gene mutations. However, the study did not demonstrate significant efficacy, with only one of five participants (20%) achieving an objective response rate (ORR). The drug was associated with adverse events, including asthenia, anemia, and decreased appetite. In a separate Phase I clinical trial (NCT01346358), LY3022855 induced a tumor-localized reduction in CD68^+^ and CD163^+^ macrophage levels in patients with solid tumors, including melanoma, as confirmed by histological analysis of biopsies. During the study, only 5.8% of patients achieved SD, while no CR or PR were reported. Notably, the study observed increases in ferritin and matrix metalloproteinases among the primary adverse effects, though the underlying mechanisms remain to be elucidated. Additionally, serious adverse events (SAEs), including pneumothorax and sepsis, were reported in 32.7% of patients [175].

In another Phase I clinical trial (NCT02718911) in patients with solid tumors, excluding melanoma, LY3022855 demonstrated superior efficacy, with a 2.8% progression-free survival rate, 29.2% ORR, and 5% CR rate in the overall population. Notably, one patient in the cohort (5%) achieved a CR. A total of 84.7% of patients experienced at least one treatment-related adverse event (TEAEs), with grade 3 TEAEs being the most prevalent (69.4%) [176]. Emactuzumab, a monoclonal antibody targeting the CSF1R receptor, has been utilized to deplete immunosuppressive TAMs in the tumor microenvironment during solid tumor therapy. Pradel et al. investigated the activity of Emactuzumab (RG7155), targeting CSF1R, in a C57BL/6 transgenic mouse model carrying BRAF and PTEN mutations. Results showed a decrease in CD163^+^ TAMs and an increase in CD8^+^ T-lymphocyte infiltration into the tumor. Additionally, a decline in CD206^+^ macrophages, which are implicated in angiogenesis, was observed, accompanied by a decrease in microvessel density and reduced VEGF expression in tumor tissue. These changes contributed to a substantial reduction in tumor volume and the number of metastases [177].

Ongoing clinical trials (NCT05417789, NCT02323191, NCT02760797, NCT01494688, NCT02923739) are investigating the use of emactuzumab in various tumor types, excluding melanoma. The results suggest that while emactuzumab effectively reduces TAMs; this alone is insufficient to achieve a clinically significant antitumor effect without additional therapy [178].

#### CAR-T

An alternative approach involves the use of CAR-T-cells to eliminate TAMs from melanoma stroma. Rodriguez-Garcia et al. engineered CAR-T-cells specific to folate receptor β (FRβ), which is expressed in a subpopulation of M2-like TAMs. In their study, the authors successfully depleted FRβ^+^ TAMs in syngeneic C57BL/6 mouse models of melanoma (B16-F10 lineage). This resulted in a reduction in TAM numbers, suppression of angiogenesis, and increased infiltration of T-cells into the tumor stroma, leading to subsequent tumor regression. The treatment also caused a reduction in tumor size, delayed metastasis, and improved survival of the animals. Furthermore, the therapeutic regimen led to a decrease in the levels of proangiogenic factors, such as VEGF and IL-10, thereby validating its impact on the TME [179].

#### Repolarization

Another promising approach for TAM target therapy is the repolarization of the phenotype from M2- to M1-like [180]. A study by Banerjee et al. demonstrated a synergistic effect of an immunomodulator derived from *Mycobacterium indicus pranii* in repolarizing TAM, in combination with a DTA-1 antibody against glucocorticoid-induced TNF receptor family related protein (GITR), which plays a pivotal role in the immunological tolerance provided by CD4^+^ CD25^+^ Treg cells. Notably, neither the immunomodulator nor DTA-1 alone demonstrated a prolonged suppressive effect on B16F10 melanoma cells in syngeneic C57BL/6 mouse models. The observed synergistic effect was found to be dependent on the transient nature of repolarization towards the M1-phenotype, which may not withstand the suppressive influence of Treg cells. Furthermore, the application of the DTA-1 antibody alone led to a modest depletion of the Treg cell population [181]. The repolarization of TAMs within the melanoma microenvironment can be approached through the combination of complex chemo- and immunotherapy [182], polymeric nanoparticles with cytokines [183] or mRNA [184], small-molecule inhibitors of signaling pathways [155], monoclonal antibodies against pattern recognition receptors [185], as well as various immunomodulator molecules [186] and bioactive inhibitor peptides [187].

TAMs are key components of the TME that play a pivotal role in tumor progression. However, the heterogeneity within this population suggests that macrophage plasticity could shift their function towards an anti-angiogenic, tumoricidal direction. Despite various strategies aimed at reducing the number of TAMs or altering their phenotype, successful clinical outcomes remain limited due to the heterogeneity of TAMs and the difficulty of targeting them. As a result, there is an urgent need for the development of combination therapies that incorporate targeting agents, immunotherapy, and conventional anticancer drugs to optimize treatment outcomes. This remains a critical area of focus in ongoing clinical trials. Importantly, the field has seen the emergence of certain anti-TAM therapies that enhance traditional methods of targeting different tumors, highlighting the potential for continued research in this area. All clinical data (Table 3) and preclinical data (Table 4) summarized below.

**Table 3:** Clinical researches of TAM-targeted melanoma therapy

Target	Type of drug	Additional terms	Clinical trials ID	Phase	Disease	Status/Results	Reference
CSF1R	PLX3397	Pembrolizumab	NCT02452424	I/IIa	Progressive Melanoma	**Terminated, not recruiting.** Two of the 13 participants had a partial response. 11 had progressive or stable disease.	–
		–	NCT02071940	II	Progressive acral and mucosal melanoma with mutation in *KIT* gene	**Terminated, not recruiting.** Results not published.	–
	LY3022855	–	NCT03101254	I/II	Progressive Melanoma with mutation in *BRAF* gene	**Completed.** Did not show significant efficacy, with ORR observed in only 1 of 5 participants (20%). Common adverse events included asthenia, anemia and decreased appetite.	–
		–	NCT01346358	I	Solid Tumors (including melanoma)	**Completed.** The drug reduced certain macrophage populations. Only 5.8% of patients achieved SD, with no CR or PR. AEs increased, with 32.7% experiencing serious events, including pneumothorax and sepsis; mechanisms remain unclear.	[175]
		–	NCT02718911	I	Solid Tumors (without melanoma)	**Completed.** The incidence of PR and SD was 2.8%, 29.2% and in the general population, respectively. One patient in the cohort (5%) achieved CR. Among all patients, 84.7% experienced at least one TEAE, with grade 3 being the most frequent (69.4%).	[176]
	Emactuzumab	–	NCT05417789	III	Solid Tumours (without melanoma)	**Active, not recruiting.** Results not published.	–
		Atezolizumab	NCT02323191	I	Solid Tumors (without melanoma)	**Completed.** Results not published.	–
		RO7009789	NCT02760797	I	Solid Tumors (without melanoma)	**Completed.** Results not published.	–
		Paclitaxel	NCT01494688	I	Solid Tumors (without melanoma)	**Completed.** Results not published.	–
		Paclitaxel and bevacizumab	NCT02923739	II	Solid Tumors (without melanoma)	**Terminated, not recruiting.** Effectively reduces TAMs, but this is not sufficient to achieve a clinically significant antitumor effect.	–

Note: Abbreviations: CSF1R—colony-stimulating factor 1 receptor, ORR—overall response rate, CR—complete response, PR—partial response, SD—stable disease, TEAE—treatment-emergent adverse event, TAM—tumor-associated macrophages.

**Table 4:** Preclinical researches of TAM-targeted melanoma therapy

Target	Type of drug	Additional terms	Research object	Results	Reference
TAMs	Trabectedin	–	Syngeneic B16-BL6 and K1735-M2 melanoma cell lines in C57BL/6 and C3H/HeN mice *in vivo*	Inhibited melanoma growth and reduced tumor blood vessel density and TAMs.	[154]
	Dichloromethylene-bisphosphonate or Clodronate – hydrophilic bisphosphonate	–	Mouse models with solid tumors *in vivo*	Depleted TAMs and significantly suppressed tumor growth and vascularization.	[162]
		–	SK-Mel-5 and UACC-62 melanoma cell lines *in vitro*; xenografted SK-Mel-5 melanoma cell line in athymic nude mice *in vivo*	Inhibited tumor growth without systemic toxicity.	[163]
	Clo-Lipo-DOTAP	–	Syngeneic B16/F10 melanoma cell line in C57BL/6JOlaHsd mice *in vivo*	Reduced primary tumors and lung metastases; decreased angiogenic cytokines; no effect on Ki-67 expression.	[164]
		–	Xenografted IIB-MEL-J melanoma cell line in athymic NIH(S)-nu/nu mice *in vivo*	Reduced tumor size, TAM infiltration, angiogenesis, and endothelial markers; suppressed MCP-1^+^ melanoma and macrophage viability.	[165]
	Immunomodulator from *Mycobacterium indicus pranii*	DTA-1 antibody	Syngeneic B16-F10 melanoma cell line in C57BL/6 mice *in vivo*	Repolarization to M1 was transient; DTA-1 slightly depleted Tregs, limiting sustained effect.	[181]
CSF1R	PLX3397	Immunotherapy	Syngeneic B16F10 melanoma cell line in C57BL/6 mice *in vivo*	Reduced TAM levels and enhanced CD8^+^ T-cell-mediated tumor control and IFNγ production.	[156]
		PLX51107	Syngeneic YUMM1.7 and D4M3.A melanoma cell lines in C57BL/6 mice with BrafV600E mutation *in vivo*	PLX3397 improved response to PLX51107 in poorly responsive tumors.	[157]
	Ki20227	–	Spontaneous metastatic uveal melanoma in RETAAD mice *in vivo*	Postoperative macrophage depletion reduced tumor recurrence and improved survival.	[159]
	Emactuzumab	–	Syngeneic YUMM1.7 melanoma cell line in a C57BL/6 transgenic mice carrying BRAF and PTEN mutations *in vivo*	Reduced CD163^+^ and CD206^+^ TAMs; increased CD8^+^ T-cell infiltration; decreased tumor volume and metastasis.	[177]
С3aR	SB290157	–	Syngeneic B16-F0 and SM1WT1 melanoma cell lines in C57BL/6J mice *in vivo*	Depleted TAMs and improved survival.	[168]
CD163	Lipid nanoparticles with conjugated antibodies against CD163 loaded with doxorubicin	–	Syngeneic YUMM1.7 melanoma cell line in C57BL/6J mice and spontaneous melanoma in transgenic mice BRAFCA^/+^, PTENlox4-5^/lox4−5^ and Tg(Tyr-CreERT2) *in vivo*	Replaced suppressive CD163^+^ TAMs with immunostimulatory CD11c^high^ cells; increased CD4^+^/CD8^+^ T-cell infiltration and tumor regression.	[173]
Folate receptor β (FRβ)	CAR-T	–	Syngeneic B16-F10 melanoma cell line in C57BL/6 mice *in vivo*	Depleted FRβ^+^ TAMs; suppressed angiogenesis; increased T-cell infiltration; reduced tumor size and metastasis; improved survival.	[179]

Note: Abbreviations: TAM—tumor-associated macrophages, MCP-1—monocyte chemoattractant protein-1, Treg—regulatory T-cells, IFNγ—interferon gamma, FRβ—folate receptor beta, CSF1R—colony-stimulating factor 1 receptor.

## Conclusion

In recent years, significant progress has been made in the development of therapeutic strategies that target cells in the tumor microenvironment, including TAMs and CAFs, for melanoma treatment. Preclinical studies are exploring innovative approaches, such as liposomal delivery systems, vector molecules, and cell-based technologies. If successfully translated into clinical practice, these strategies have the potential to become breakthrough therapies for this aggressive tumor. The melanoma microenvironment, with its high invasiveness and angiogenetic potential, remains a critical target, as evidenced by vital role of TAMs and CAFs in these processes. These cells contribute to the maintenance of an inflammatory microenvironment and create favorable conditions for tumor growth, positioning them as promising targets for therapy. Despite challenges related to their phenotypic and genetic heterogeneity, TAMs and CAFs continue to be priority targets for the development of novel therapeutic approaches.

Nevertheless, the efficacy of targeted therapies in the clinic against the TME is limited by the need for combined approaches that emphasize the multifaceted effects on various microenvironment components. Modern technologies, such as single-cell RNA sequencing and spatial transcriptomics, already furnish distinctive prospects for exhaustive analysis of cell-cell interactions and TME structure. In their seminal study, Davidson et al. used scRNA-seq to investigate murine B16-F10 melanoma, unveiling the presence of 3 distinct CAF populations. The first population, designated as “immune”, manifested in early-stage tumors and was characterized by the upregulation of CSF1, CXCL12, complement C3, and C4b, playing a pivotal role in immune cell recruitment. The second population, identified as “desmoplastic”, exhibited an increased expression of ECM-related genes, contributing to fibrosis. The third population, referred to as “contractile”, was predominantly present in late-stage tumors and was marked by the expression of actin stress fiber genes [188]. In the context of human cutaneous melanoma, Lian et al. employed scRNA-seq to delineate 11 distinct cell subgroups and 3 distinct CAF subsets. Among these, CEMIP^+^ and NKD1^+^ fibroblasts were linked to EMT and potential immune therapy resistance [189]. In a related study, Xiao et al. employed imaging mass cytometry on 26 melanoma cases to identify five stromal subtypes based on marker expression: collagen^+^, FAP^+^, PDGFRβ^+^, SMA^+^, and vimentin^+^, which closely align with CAF phenotypes [190]. Du et al. integrated scRNA-seq and spatial transcriptomics across 16 distinct tumor subtypes, including acral lentiginous melanoma, and identified key stromal players and their role in the TME. For instance, the study revealed the presence of ECM-related CAFs situated at the tumor boundaries, which functioned as immune barriers, interacted with malignant cells, and regulated CD8^+^ T-cell exhaustion [191].

A comprehensive understanding of the cellular biology of stromal components and their roles in carcinogenesis and tumor progression, along with the identification of their unique signatures, is expected to facilitate the development of novel and effective therapeutic strategies that target the most critical and vulnerable elements of the melanoma microenvironment.

## Data Availability

Not applicable.
